# Vitamin D Status among Older Adults Residing in the Littoral and Andes Mountains in Ecuador

**DOI:** 10.1155/2015/545297

**Published:** 2015-08-02

**Authors:** Carlos H. Orces

**Affiliations:** Department of Medicine, Laredo Medical Center, 1700 East Saunders, Laredo, TX 78041, USA

## Abstract

*Objectives*. To estimate the prevalence of 25-hydroxyvitamin D (25(OH)D) deficiency and its determinants among older adults in Ecuador. *Methods*. 25(OH)D deficiency and insufficiency prevalence rates were examined among participants in the National Survey of Health, Wellbeing, and Aging. Logistic regression models were used to evaluate demographic characteristics associated with 25(OH)D deficiency. *Results*. Of 2,374 participants with a mean age of 71.0 (8.3) years, 25(OH)D insufficiency and deficiency were present in 67.8% (95% CI, 65.3–70.2) and 21.6% (95% CI, 19.5–23.7) of older adults in Ecuador, respectively. Women (OR, 3.19; 95% CI, 3.15–3.22), self-reported race as Indigenous (OR, 2.75; 95% CI, 2.70–2.80), and residents in rural (OR, 4.49; 95% CI, 4.40–4.58) and urban (OR, 2.74; 95% CI, 2.69–2.80) areas of the Andes Mountains region were variables significantly associated with 25(OH)D deficiency among older adults. *Conclusions*. Despite abundant sunlight throughout the year in Ecuador, 25(OH)D deficiency was significantly prevalent among older women, Indigenous subjects, and subjects residing in the Andes Mountains region of the country. The present findings may assist public health authorities to implement policies of vitamin D supplementation among older adults at risk for this condition.

## 1. Introduction

25-Hydroxyvitamin D (25(OH)D) deficiency leads to alterations in calcium and phosphorus homeostasis, resulting in secondary hyperparathyroidism with increased bone turnover, progressive bone loss, and increased risk of fractures [1–4]. Moreover, recent studies have suggested an association between 25(OH)D deficiency and poor physical performance, increased risk of falls, and mortality among older adults [5–7].

Older adults are particularly at higher risk for 25(OH)D deficiency because sunlight exposure is usually limited as a result of lifestyle changes, such as clothing and decreased outdoor activities [8]. More importantly, the concentration of 7-dehydrocholesterol in the epidermis and the total production of previtamin D_3_ after exposure to solar ultraviolet B radiation decrease considerably among older adults [9].

A previous study of 25(OH)D status among six regions in the world reported that serum 25(OH)D levels below 75 nmol/L (30 ng/mL) were prevalent in every region studied [10]. More recently, a systematic review of the prevalence of 25(OH)D deficiency in Latin America and the Caribbean concluded that 25(OH)D deficiency may be a public health problem in the region. However, the magnitude of the problem is currently unknown owing to limited number of studies in the general population [11]. In Ecuador, a small cross-sectional study conducted among older adults residing in a low income community in northwestern Quito (00°S) reported that mean 25(OH)D levels were 19.0 ng/mL [12]. Despite this evidence, there is scarce information about 25(OH)D status among older Ecuadorians. Therefore, the present study aimed to estimate the prevalence of 25(OH)D deficiency and its determinants among older adults residing in the coastal and Andes Mountains regions of the country.

## 2. Materials and Methods

The present study was based on data from participants in the National Survey of Health, Wellbeing, and Aging (Encuesta de Salud, Bienestar y Envejecimiento) conducted in 2009. This survey is a probability sample of households with a least one person aged 60 years or older residing in the Andes Mountains and coastal regions of Ecuador. In the primary sampling stage, a total of 317 sectors from rural areas (<2,000 inhabitants) and 547 sectors from urban areas of the country were selected from the 2001 population Census cartography. In the secondary sampling stage, 18 households within each sector were randomly selected based on the assumption that at least one person aged 60 years or older lives in 24% and 23% of the households in the coastal and Andes Mountains regions, respectively. Between April and August 2010, a total of 2,375 participants in the SABE II survey underwent biochemical evaluation to determine their 25(OH)D status. Survey methodology, including operation manuals, is publicly available [13].

### 2.1. Characteristics of Subjects

Age and sex were self-reported. The race of participants was classified according to the following question: “Do you consider yourself to be White, Black, Mestizo, Mulatto, or Indigenous?” Body height in centimeters and weight in kilograms were measured and the body mass index was calculated (Kg/m^2^). Subjects were asked about their living status (alone versus accompanied), region (coasts versus mountains), and area of residence (urban versus rural). Literacy was defined by answering affirmatively to the question “Can you write and read a message?”

Alcohol consumption (none, 1 day, and ≥2 days) was assessed by asking participants the following: “How many days per week on average have you drink alcohol for the past three months?” Smoking status was classified as current, former, and never. Subjects were considered to consume dairy product if they answered affirmatively to the question “Do you consume milk, cheese, or yogurt at least once per day?” Vigorous and regular physical activity was evaluated by the question, “Do you exercise such as jogging, dance, or perform rigorous physical activity at least three times weekly for the past year?” Participants who responded affirmatively were considered to engage in regular vigorous physical activity. Self-reported general health was grouped as excellent to good or fair to poor.

The following activities of daily living (ADLs) were included in the present study: walking across a room, dressing, bathing, eating, getting in and out of bed, and using the toilet. Those participants who needed help or were unable to perform one or more of the ADLs as a result of health problems were considered functionally impaired. Similarly, participants who reported difficulty walking a few city blocks or walking up a flight of stairs were considered to have mobility disability.

Serum 25(OH)D was measured by liquid chromatography at NetLab laboratory (Quito, Ecuador). The lowest limit of detection for the serum 25(OH)D assay was 4 ng/mL. 25(OH)D status was classified as <20 ng/mL and <30 ng/mL, which are cut-off values recommended by the Institute of Medicine and the Endocrine Society to define vitamin D deficiency and insufficiency, respectively [14, 15]. One subject with a toxic 25(OH)D level (>150 ng/mL) was excluded from this analysis [3].

### 2.2. Statistical Analysis

ANOVA and *t*-tests for continuous variables and the chi-square test for categorical variables were used to compare mean 25(OH)D levels and the proportions of vitamin D deficiency and insufficiency, respectively. Logistic regression models adjusted for age, sex, and body mass index were created to examine the independent associations between sociodemographic, behavior, and health characteristics of the participants and 25(OH)D deficiency. Results of the multivariate model are presented as odds ratios (OR) with their 95% confidence intervals (95% CI). To examine the geographic distribution of 25(OH)D deficiency in Ecuador, the proportion of 25(OH)D deficiency by provinces of the coastal and Andes Mountains regions of the country was age-adjusted by the direct method using the 2010 Census population of Ecuador as the standard [16]. All analyses used sample weights to account for the complex survey design. Statistical analyses were performed using SPSS, version 17 software (SPSS Inc., Chicago, IL).

## 3. Results

A total of 2,374 participants with a mean age of 71.0 (8.3) years had 25(OH)D measured.  Table 1 shows the characteristics of participants and their mean 25(OH)D levels. In general, 25(OH)D levels decreased with advancing age and were lower among women. By race, 25(OH)D levels were significantly lower among Indigenous as compared to other ethnic groups. Moreover, participants who engaged in regular vigorous physical activity and consumed dairy products had higher 25(OH)D levels than those who did not. On the contrary, lower 25(OH) levels were seen among subjects who never smoked or drink alcohol, and subjects with self-reported mobility and ADLs limitations.

Overall, 67.8% (95% CI, 65.3–70.2) of participants had 25(OH)D levels below 30 ng/mL and 21.6% (95% CI, 19.5–23.7) had levels below 20 ng/mL, representing an estimated 808,000 and 256,000 older Ecuadorians with 25(OH)D insufficiency and deficiency, respectively.  Table 2 shows the prevalence of 25(OH)D deficiency and insufficiency according to certain characteristics of the survey participants. In general, 25(OH)D deficiency and insufficiency were considerably higher among the elderly, women, Indigenous subjects, obese subjects, and residents in rural and urban areas of the Andes Mountains.

**Figure 1:**
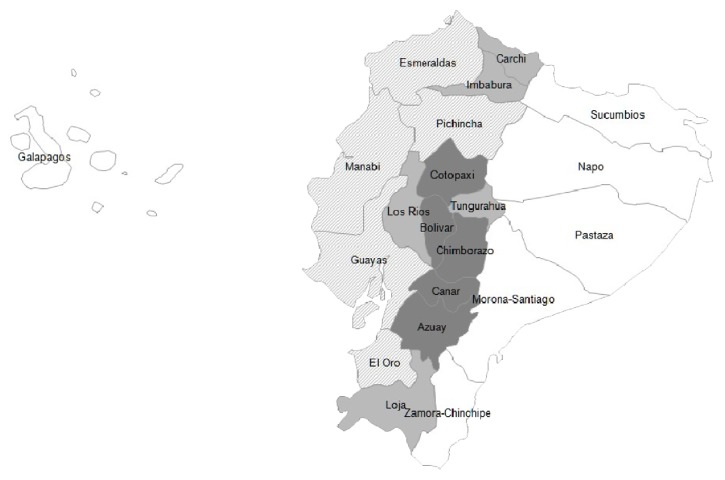
Prevalence of vitamin D deficiency among older adults in Ecuador.

As shown in Figure 1, the age-adjusted prevalence of 25(OH)D deficiency among older adults varied across the country. However, residents in provinces located in the Andes Mountains region had consistently higher 25(OH)D deficiency prevalence rates than those residing in provinces along the coastal region. For instance, up to 62.0% and 53.8% of residents in the provinces of Bolivar and Chimborazo were considered deficient in 25(OH)D. Conversely, low prevalence of 25(OH)D deficiency ranging from 8.3% to 17.8% was found among subjects residing in provinces of the coastal region.

As shown in Table 3, the results of the multivariate logistic regression model indicate that women (OR, 3.19; 95% CI, 3.15–3.22), self-reported race as Indigenous (OR, 2.75; 95% CI, 2.70–2.80), and subjects residing in rural (OR, 4.49; 95% CI, 4.40–4.58) and urban (OR, 2.74; 95% CI, 2.69–2.80) areas of the Andes Mountains region were variables strongly and independently associated with 25(OH)D deficiency among older adults in Ecuador.

## 4. Discussion

The present study indicates that 25(OH)D deficiency and insufficiency were present in 21.6% and 67.8% of older adults in Ecuador, respectively. Moreover, the prevalence of 25(OH)D deficiency was particularly common among women, Indigenous subjects, and residents in the Andes Mountains region of the country.

Compared with studies of 25(OH)D status among postmenopausal women in Latin America, the prevalence of 25(OH)D deficiency reported among women in Santiago (33°S), Chile, was higher than that found among women in Ecuador [17]. Similarly, a study of ambulatory adults aged 65 years or older in seven cities from Argentina demonstrated that the prevalence of 25(OH)D deficiency varied from 73% in the southern cities (41° to 55°S) to 50% in the northern cities (26°S to 27°S) [18]. On the contrary, the prevalence of 25(OH)D deficiency among women with osteopenia and osteoporosis across six cities in Brazil was lower than that found among Ecuadorian women [19]. It is of interest that, among postmenopausal Brazilian women, an inverse correlation was found between the mean serum 25(OH)D level site and latitude with a mean reduction of 0.28 ng/mL for each latitude's degree south of the equatorial line. Despite these findings, only 10% of postmenopausal women from the most northern city of Recife, Brazil (8°S), had evidence of 25(OH)D deficiency [19].

Although there is scarce data about 25(OH)D status among older men in Latin America, the prevalence of 25(OH)D deficiency among older men in Ecuador was lower than that reported among older men in Recife (8°S), Brazil, and subjects who participated in the Osteoporotic Fractures in Men Study [20, 21].

It is of relevance that a high prevalence of 25(OH)D deficiency was found among participants who self-reported their race as Indigenous. Although Indigenous people accounted for only 7.0% of the population in Ecuador in 2010, 25(OH)D deficiency was present in up to 40.5% of older adults from this ethnic group [16]. In fact, after adjustment for age, sex, and BMI, the prevalence of 25(OH)D deficiency was 2.7-fold higher among older Indigenous subjects as compared with Whites. According to results from the National Census 2010, the Indigenous population is predominantly concentrated in certain provinces of the Andes Mountains region such as Chimborazo, Cotopaxi, Imbabura, and Bolivar [16]. It is of relevance that higher prevalence rates of 25(OH)D deficiency were also found among older residents in these provinces.

Despite of abundant sunlight exposure in the country, it is conceivable that the combination of dark skin pigmentation, clouds cover, and wearing wool ponchos to protect from cold climates at high altitude may be an effective barrier to ultraviolet B radiation and account for the high prevalence of 25(OH)D deficiency observed among older Indigenous people in Ecuador. Previous studies have shown a high prevalence of 25(OH)D deficiency and insufficiency in young healthy Jordanian women and ultra-Orthodox men in Israel, whose dress-code precludes sunlight exposure [22, 23]. Likewise, consistent with the present findings, a recent investigation reported a high prevalence of 25(OH)D deficiency and insufficiency among Indigenous children living at high altitudes in San Antonio de Los Cobres (24°S), Argentina [24].

It is of interest that older Blacks had the lowest prevalence of 25(OH)D deficiency in Ecuador. This finding may be partly explained by the fact that 72.7% of subjects from this racial group reside in the coastal region characterized by warm climates and abundant sunlight exposure throughout the year [16]. Therefore, it is feasible that older Blacks devote more time to outdoor physical activities resulting in prolonged exposure to ultraviolet B radiation. For instance, the province of Esmeraldas has the highest proportion of Blacks in the country. Conversely, this province had the lowest age-adjusted prevalence of 25(OH)D deficiency among older adults in the country. These results contrast with those from previous studies, which have consistently reported higher prevalence rates of 25(OH)D deficiency among Blacks [25, 26].

The prevalence of 25(OH)D deficiency among older Ecuadorians varied considerably among regions and areas of the country. However, subjects residing in rural and urban areas of the Andes Mountains had 4.4- and 2.7-fold higher rates of 25(OH)D deficiency as compared with residents in rural areas of the coastal region, respectively. This marked geographic disparity in the prevalence of 25(OH)D deficiency among older Ecuadorians deserves attention and should be further investigated. However, it may be partly explained by skin phenotype, limited outdoor activities, and cultural clothing as a result of cool temperatures among subjects living in the Andes Mountains region of the country. Moreover, at similar latitudes, higher prevalence rates of 25(OH)D deficiency were found among subjects residing in the Andes Mountains region. For instance, the age-adjusted prevalence of 25(OH)D deficiency in the Andes Mountains province of Carchi (2°2′N) was 20.4%. On the contrary, the prevalence of 25(OH)D deficiency in the coastal province of Esmeraldas (0°58′S) was only 8.3%.

Older Ecuadorians who engaged in regular vigorous physical activity had higher levels of 25(OH)D as compared to those who did not. Similarly, Scragg and Camargo Jr. reported a significant relationship between the frequency of regular outdoor physical activity and higher levels of 25(OH)D. It is of interest that the authors also found similar 25(OH)D levels between subjects aged 20–39 and 60 or more years who engaged frequently in outdoors activities [27]. Moreover, a recent analysis of the Longitudinal Aging Study Amsterdam demonstrated that outdoor physical activity with a high intensity, such as gardening and cycling, was associated with higher 25(OH)D levels [28]. Maintaining an adequate 25(OH)D status is a major public health task because low 25(OH)D concentrations among older adults have been associated with poor muscle strength, worse physical performance measures, disability, hip fractures, and mortality [5, 29, 30].

Several limitations should be mentioned in interpreting the present results. First, participants self-reported their characteristics, which may be a source of recall bias. Second, dietary intake of vitamin D or use of vitamin D supplements was not assessed in the survey. Third, the laboratory did not report the 25(OH)D intra- and interassay coefficient of variation. Fourth, these results may be generalized to older adults residing in the coastal and Andes Mountains regions of the country. However, older adults from the Amazon region and the Galapagos Islands represent only 3.3% of the population aged 60 years or older in Ecuador [31]. Despite these limitations, the present study is the first to report the prevalence of 25(OH)D deficiency among older Ecuadorians.

In conclusion, despite abundant sunlight throughout the year in Ecuador, 25(OH)D deficiency was significantly prevalent among older women, Indigenous subjects, and residents in the Andes Mountains region of the country. The present findings may assist public health authorities to implement policies of vitamin D supplementation, particularly among older adults at risk for this condition.

## Figures and Tables

**Table 1:** 25(OH)D levels by selected characteristics among older adults in Ecuador.

	Subjects (%)	25(OH)D levels (SD)	*P* value
Age groups, yrs.			
60–69	1,158 (51.7)	27.6 (11.5)	<0.001
70–79	724 (31.4)	25.5 (9.7)	
≥80	403 (16.8)	25.9 (13.2)	
Sex			
Men	1,072 (44.5)	30.0 (12.3)	<0.001
Women	1,302 (55.5)	23.8 (9.4)	
Race			
Indian	207 (10.7)	21.6 (8.8)	<0.001
Black	80 (3.6)	27.7 (12.4)	
Mestizo	1,584 (69.8)	26.9 (10.6)	
Mulatto	84 (3.5)	26.4 (8.4)	
White	273 (12.5)	26.7 (12.3)	
BMI (Kg/m^2^)			
Underweight	63 (2.3)	26.6 (11.6)	<0.001
Normal	920 (41.9)	28.0 (12.3)	
Overweight	877 (37.7)	26.1 (10.1)	
Obese	408 (18.1)	24.9 (10.7)	
Living alone			
Yes	209 (9.1)	27.1 (13.1)	<0.001
No	2,165 (90.5)	26.5 (11.0)	
Area of residence			
Rural mountains	505 (20.4)	21.7 (8.0)	<0.001
Urban mountains	685 (29.7)	22.8 (6.2)	
Rural coast	505 (13.1)	32.6 (14.1)	
Urban coast	877 (36.8)	30.0 (12.5)	
Literacy			
Yes	1,664 (69.4)	27.1 (10.8)	<0.001
No	707 (30.6)	25.2 (12.1)	
Consume dairy products			
Yes	1,586 (66.8)	27.0 (11.9)	<0.001
No	788 (33.2)	25.5 (9.7)	
Smoking status			
Current	243 (10.3)	28.9 (9.8)	<0.001
Former	661 (27.9)	29.6 (13.1)	
Never	1,464 (61.8)	24.6 (10.1)	
Alcohol use			
None	1,891 (77.9)	26.0 (11.0)	<0.001
1 day	420 (19.4)	27.8 (11.6)	
≥2 days	60 (2.7)	31.7 (12.6)	
Physical activity			
Yes	747 (32.9)	27.7 (11.4)	<0.001
No	1,626 (67.1)	26.0 (11.1)	
Self-reported health			
Excellent to good	557 (24.6)	26.3 (11.0)	<0.001
Fair to poor	1,812 (75.4)	26.6 (11.3)	
Mobility disability			
Yes	990 (41.4)	25.5 (10.4)	<0.001
No	1,384 (58.6)	27.2 (11.7)	
Limitation in ADLs			
Yes	652 (26.5)	25.9 (11.9)	<0.001
No	1,719 (73.5)	26.7 (11.0)	

**Table 2:** Prevalence of 25(OH)D deficiency and insufficiency among older adults in Ecuador.

	25(OH)D < 20 ng/mL % (95% CI)	*P* value	25(OH)D < 30 ng/mL % (95% CI)	*P* value
Age groups, yrs.				
60–69	18.2 (15.6–21.1)	<0.001	64.8 (61.2–68.3)	<0.001
70–79	22.4 (18.9–26.3)		70.5 (66.1–74.6)	
≥80	28.5 (22.7–35.0)		68.7 (62.4–74.5)	
Sex				
Men	11.5 (9.4–13.9)	<0.001	55.9 (52.1–59.7)	<0.001
Women	29.6 (26.5–32.9)		77.4 (74.2–80.2)	
Race				
Indian	40.5 (31.3–50.4)	<0.001	84.0 (76.2–89.6)	<0.001
Mestizo	19.2 (17.1–21.6)		65.8 (62.7–68.8)	
Black	16.0 (9.2–26.4)		63.0 (49.7–74.5)	
Mulatto	15.3 (8.0–27.1)		69.4 (54.7–80.9)	
White	22.5 (17.1–29.1)		67.9 (60.6–74.3)	
BMI (Kg/m^2^)				
Underweight	20.4 (11.1–34.5)	<0.001	65.9 (49.9–79.0)	<0.001
Normal	19.3 (16.3–22.8)		61.6 (57.3–65.7)	
Overweight	19.2 (16.3–22.6)		71.1 (67.4–74.6)	
Obesity	28.3 (23.3–33.9)		74.0 (68.1–79.2)	
Living status				
Alone	20.9 (15.3–27.8)	<0.001	69.1 (60.0–77.0)	<0.001
Accompanied	21.6 (19.5–23.9)		67.7 (65.1–70.2)	
Area of residence				
Rural mountains	34.8 (29.3–40.8)	<0.001	86.6 (82.5–89.9)	<0.001
Urban mountains	26.6 (22.8–30.8)		86.2 (82.5–89.2)	
Rural coast	10.6 (7.1–15.6)		44.3 (37.4–51.5)	
Urban coast	14.0 (11.6–16.9)		50.9 (46.7–55.2)	
Literacy				
Yes	19.2 (17.0–21.5)	<0.001	65.7 (62.7–68.5)	<0.001
No	27.1 (22.8–31.8)		73.2 (68.7–77.2)	
Consume dairy products				
Yes	21.0 (18.6–23.6)	<0.001	66.9 (63.9–69.8)	<0.001
No	22.8 (19.0–27.0)		69.7 (65.1–73.8)	
Smoking status				
Current	13.5 (8.6–20.6)	<0.001	54.3 (46.0–62.4)	<0.001
Former	14.3 (11.3–17.8)		57.7 (52.8–62.4)	
Never	26.4 (23.6–29.4)		75.0 (72.0–77.8)	
Alcohol use				
None	22.8 (20.5–25.4)	<0.001	68.8 (66.0–71.4)	<0.001
1 day	17.9 (13.7–23.0)		66.2 (60.2–71.8)	
≥2 days	11.3 (4.8–24.3)		51.7 (34.8–68.2)	
Physical activity				
Yes	18.8 (15.6–22.5)	<0.001	65.5 (60.8–69.9)	<0.001
No	22.9 (20.4–25.6)		69.0 (66.0–71.8)	
Self-reported health				
Excellent to good	22.5 (18.6–27.0)	<0.001	70.6 (65.9–74.9)	<0.001
Fair to poor	21.1 (18.8–23.6)		66.8 (63.9–69.6)	
Mobility disability				
Yes	22.9 (19.6–26.4)	<0.001	69.8 (65.9–73.5)	<0.001
No	20.6 (18.1–23.4)		66.4 (63.1–69.5)	
Limitation in ADLs				
Yes	25.4 (21.2–30.0)	<0.001	67.1 (62.1–71.6)	<0.001
No	20.2 (17.9–22.7)		68.1 (65.2–70.9)	

**Table 3:** Associations between characteristics of participants and 25(OH)D deficiency.

	Crude OR (95% CI)	Adjusted OR (95% CI)
Age groups, yrs.		
60–69	1.00	1.00
70–79	1.30 (1.28–1.31)	1.31 (1.30–1.33)
≥80	1.79 (1.77–1.81)	1.54 (1.52–1.56)
Sex		
Men	1.00	1.00
Women	3.25 (3.21–3.28)	3.19 (3.15–3.22)
BMI (Kg/m^2^)		
Underweight	1.00	1.00
Normal	0.93 (0.90–0.96)	0.99 (0.95–1.02)
Overweight	0.92 (0.89–0.95)	0.92 (0.89–0.95)
Obesity	1.53 (1.48–1.58)	1.30 (1.25–1.34)
Race		
Indigenous	2.34 (2.30–2.38)	2.75 (2.70–2.80)
Black	0.65 (0.63–0.67)	0.75 (0.85–0.88)
Mestizo	0.81 (0.80–0.83)	0.89 (0.88–0.91)
Mulatto	0.62 (0.60–0.63)	0.71 (0.68–0.73)
White	1.00	1.00
Area of residence		
Rural mountains	4.51 (4.43–4.59)	4.49 (4.40–4.58)
Urban mountains	3.05 (3.00–3.11)	2.74 (2.69–2.80)
Urban coast	1.37 (1.35–1.40)	1.22 (1.20–1.25)
Rural coast	1.00	1.00
Living status		
Alone	1.04 (1.03–1.06)	1.00 (0.98–1.01)
Accompanied	1.00	1.00
Literacy		
Yes	1.00	1.00
No	1.56 (1.54–1.57)	1.16 (1.15–1.17)
Consume dairy products		
Yes	1.00	1.00
No	1.10 (1.09–1.11)	1.14 (1.13–1.15)
Smoking status		
Current	0.43 (0.42–0.44)	0.97 (0.95–0.99)
Former	0.46 (0.45–0.46)	0.88 (0.87–0.89)
Never	1.00	1.00
Alcohol use		
None	1.00	1.00
1 day	0.73 (0.72–0.74)	1.20 (1.18–1.22)
≥2 days	0.43 (0.41–0.44)	0.87 (0.84–0.91)
Physical activity		
Yes	1.00	1.00
No	0.78 (0.77–0.78)	1.15 (1.13–1.16)
Self-reported health		
Excellent to good	1.00	1.00
Fair to poor	0.92 (0.91–0.92)	0.73 (0.72–0.74)
Mobility disability		
Yes	1.13 (1.12–1.14)	0.75 (0.74–0.76)
No	1.00	1.00
Limitations in ADLs		
Yes	1.34 (1.33–1.35)	1.01 (1.00–1.02)
No	1.00	1.00
